# Long-Term Mortality after New-Onset Atrial Fibrillation in COVID-19

**DOI:** 10.3390/jcm12082925

**Published:** 2023-04-18

**Authors:** Stjepan Jurisic, Mathis Komminoth, Atanas Todorov, Daniela A. Bertschi, Martin Jurisic, Ivica Vranjic, Benedikt Wiggli, Hansruedi Schmid, Catherine Gebhard, Caroline E. Gebhard, Bettina Heidecker, Jürg-Hans Beer, Dimitri Patriki

**Affiliations:** 1 Department of Internal Medicine, Cantonal Hospital of Baden, 5404 Baden, Switzerland; 2 Department of Cardiology, University Hospital of Zurich, 8091 Zurich, Switzerland; 3 Department of Nuclear Medicine, Cardiac Imaging, University Hospital Zurich, 8091 Zurich, Switzerland; 4 Department of Cardiovascular Diseases, German Heart Centre Munich, Technical University Munich, 80636 Munich, Germany; 5 Department of Infectious Diseases & Infection Control, Cantonal Hospital of Baden, 5404 Baden, Switzerland; 6 Center for Molecular Cardiology, University of Zurich, 8952 Schlieren, Switzerland; 7 Intensive Care Unit, Department of Acute Medicine, University Hospital Basel, University of Basel, 4031 Basel, Switzerland; 8 Deutsches Herzzentrum der Charité, Universitätsmedizin Berlin, Hindenburgdamm 30, 12203 Berlin, Germany

**Keywords:** COVID-19, atrial fibrillation, outcome

## Abstract

**Background**: Atrial fibrillation (AF) has been described as a common cardiovascular manifestation in patients suffering from coronavirus disease 2019 (COVID-19) and has been suggested to be a potential risk factor for a poor clinical outcome. **Methods**: In this observational study, all patients hospitalized due to COVID-19 in 2020 in the Cantonal Hospital of Baden were included. We assessed clinical characteristics, in-hospital outcomes as well as long-term outcomes with a mean follow-up time of 278 (±90) days. **Results**: Amongst 646 patients diagnosed with COVID-19 (59% male, median age: 70 (IQR: 59–80)) in 2020, a total of 177 (27.4%) patients were transferred to the intermediate/intensive care unit (IMC/ICU), and 76 (11.8%) were invasively ventilated during their hospitalization. Ninety patients (13.9%) died. A total of 116 patients (18%) showed AF on admission of which 34 (29%) had new-onset AF. Patients with COVID-19 and newly diagnosed AF were more likely to require invasive ventilation (OR: 3.5; *p* = 0.01) but did not encounter an increased in-hospital mortality. Moreover, AF neither increased long-term mortality nor the number of rehospitalizations during follow-up after adjusting for confounders. **Conclusions**: In patients suffering from COVID-19, the new-onset of AF on admission was associated with an increased risk of invasive ventilation and transfer to the IMC/ICU but did not affect in-hospital or long-term mortality.

## 1. Introduction

The occurrence of arrhythmias in patients suffering from coronavirus disease 2019 (COVID-19) is associated with fatal outcomes and poor clinical trajectory [1,2,3,4,5,6,7]. Multiple studies reported an incidence of arrhythmias varying from 2% to 34% in patients with COVID-19 [8,9,10,11,12,13,14,15,16,17,18]. Atrial fibrillation (AF) is the most common arrhythmia in patients with COVID-19 and is associated with increased mortality [19,20]. Inflammation is a relevant trigger of AF, potentially via altered electrophysiology due to structural and electrical atrial remodeling [21,22]. Accordingly, it was hypothesized that these alterations might occur in patients suffering from COVID-19. Recent studies in patients with COVID-19 suggest that AF may worsen in-hospital outcomes as compared to those without AF [23,24,25,26]. The latter is crucial, as periods of a high incidence of COVID-19 lead to the necessity of fast triaging of potentially high-risk cases. However, there are conflicting results in the literature with regard to the prevalence of atrial fibrillation in COVID-19 and its association with clinical trajectory [23]. Moreover, to the best of our knowledge, the impact of new-onset AF in COVID-19 on long-term outcomes has not been evaluated as of today. Hence, we sought to investigate the clinical characteristics, prognostic implications, and long-term effects of AF in patients with COVID-19.

## 2. Methods

This is a retrospective single-center observational study. The study protocol was reviewed by the local ethics committee (BASEC: 2020-00952). We systematically included all patients over 18 years of age who were hospitalized at the Cantonal Hospital Baden due to COVID-19 in 2020. All participants were tested positive for severe acute respiratory syndrome coronavirus type 2 (SARS-CoV-2) via polymerase chain reaction (PCR). Patient records were reviewed and data on clinical profiles and outcomes were collected from January 2020 until December 2020 during the first phase of the pandemic. Electrocardiograms (ECG) were obtained on admission in all patients with COVID-19. Two experienced physicians evaluated all ECGs on admission and screened for AF with diagnostic criteria based on the European Society of Cardiology guidelines [27]. In cases of disagreement, a third reviewer was included in finalizing the diagnosis. Patients were then clustered into patients with new-onset AF, patients with known AF on admission or patients without AF on admission. Follow-up was performed after a minimum of 60 days. Patients, their families, or family doctors were contacted via telephone to gain information about the patient’s clinical condition. In order to put each of our COVID-19 hospitalizations in perspective to the epidemiological situation and hospital capacity, we also implemented the incidence and prevalence of COVID-19 cases in the catchment area of our hospital using official data from “Bundesamt für Gesundheit” in Switzerland (https://www.covid19.admin.ch/de/epidemiologic/case (accessed on 1 May 2021)).

### 2.1. Endpoints

To evaluate the association of AF on admission in patients with COVID-19, we evaluated the clinical trajectory of all patients during hospitalization. Our primary endpoints were in-hospital death from any cause, transfer to the IMC/ICU and need of invasive ventilation during hospitalization. Our secondary endpoints were long-term outcomes defined as all-cause out-of-hospital death after hospitalization and number of rehospitalizations for any cause within the follow-up period.

### 2.2. Statistical Analysis

**Figure 1:**
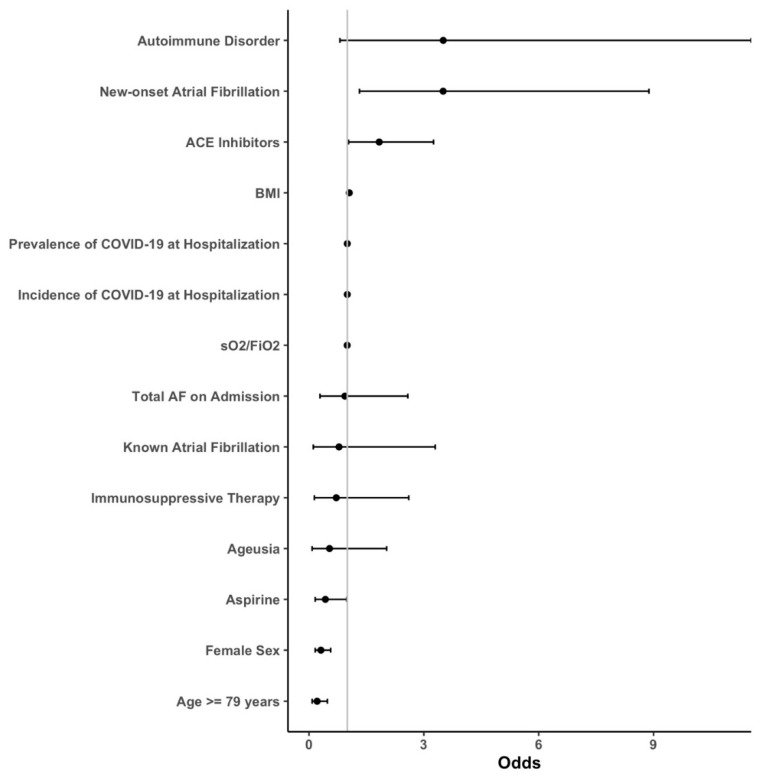
Forest plots of the primary outcome “Invasive Ventilation”. The risk of invasive ventilation was assessed by means of sequential logistic models with a stepwise reduction based on the Aikake information criterion. Abbreviations: ACE = angiotensin-converting enzyme; AF = atrial fibrillation; BMI = body mass index; sO_2_ = oxygen saturation; FiO_2_ = fraction of inspired oxygen; COVID-19 = coronavirus disease 2019.

**Figure 2:**
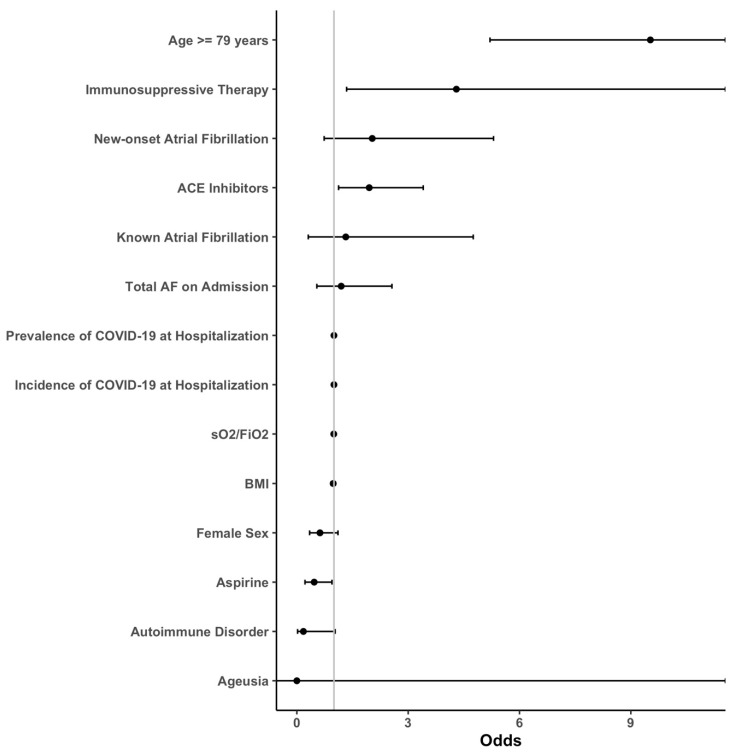
Forest plots of the primary outcome “In-Hospital Death”. The transfer risk for in-hospital death was assessed by sequential logistic models with a stepwise reduction based on the Aikake information criterion. Abbreviations: ACE = angiotensin-converting enzyme, AF = atrial fibrillation, BMI = body mass index, sO_2_ = oxygen saturation, FiO_2_ = fraction of inspired oxygen, COVID-19 = coronavirus disease 2019.

**Figure 3:**
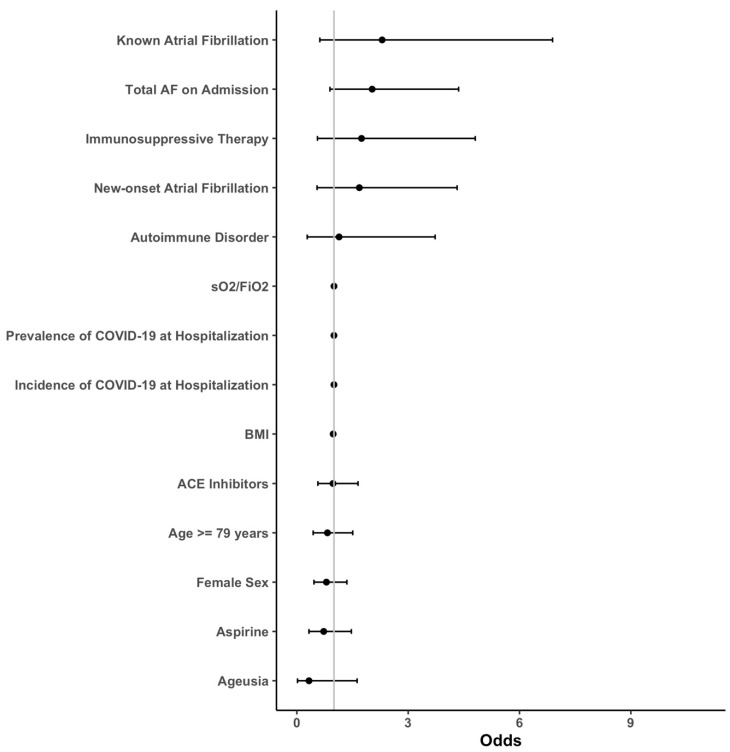
Forest plots of the secondary outcome “Repeated Hospitalization”. The transfer risk for repeated hospitalization was assessed by sequential logistic models with a stepwise reduction based on the Aikake information criterion. Abbreviations: ACE = angiotensin-converting enzyme; AF = atrial fibrillation; BMI = body mass index; sO_2_ = oxygen saturation; FiO_2_ = fraction of inspired oxygen; COVID-19 = coronavirus disease 2019.

**Figure 4:**
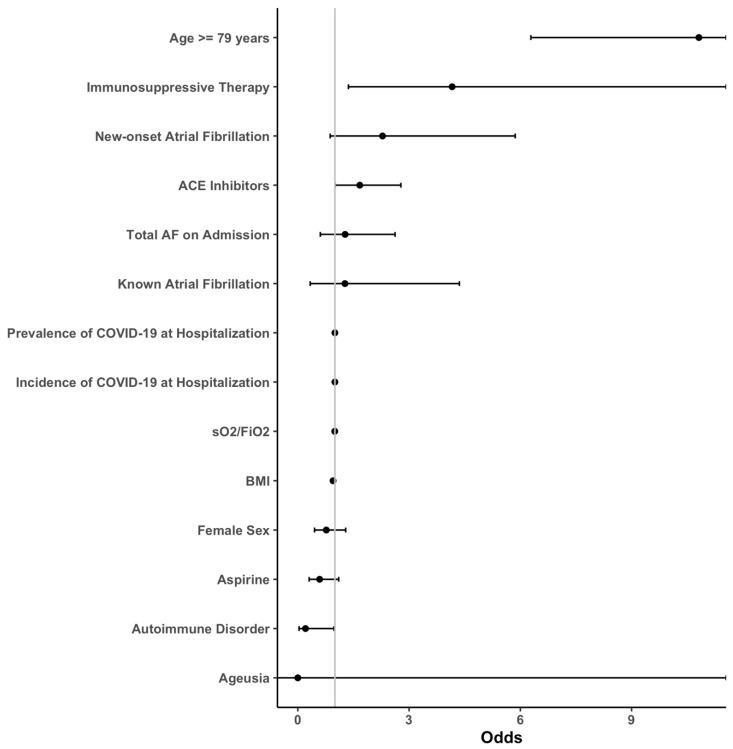
Forest plots of the secondary outcome “Long-Term Mortality”. The transfer risk for long-term mortality was assessed by sequential logistic models with a stepwise reduction based on the Aikake information criterion. Abbreviations: ACE = angiotensin-converting enzyme; AF = atrial fibrillation; BMI = body mass index; sO_2_ = oxygen saturation; FiO_2_ = fraction of inspired oxygen; COVID-19 = coronavirus disease 2019.

Descriptive statistics were used to characterize clinical data, baseline parameters, and laboratory measurements. The initially formed groups of patients with and without new-onset AF, as well as patients with known AF, were then analyzed and compared. Nominal variables were compared using the chi-square test, whereas metric scaled and normally distributed variables were reported as mean with standard deviation and analyzed with the Student’s *t*-test. Otherwise, median and interquartile range (IQR) was given and the Mann–Whitney U-test was performed. An age cut-off for in-hospital death was calculated using Receiver Operating Characteristic (ROC) and calculated Youden Index. For multivariate analysis of each outcome, sequential logistic models were built using either (1) no covariates, (2) AF grouping as the single covariate, (3) AF and known confounders ((age, body mass index (BMI), known cardiomyopathy (CMP), known coronary artery disease (CAD), chronic airway disease, renal insufficiency, diabetes, arterial hypertension and sex) as covariates or (4) a full model including all available covariates. If any of the models including covariates were significantly different from the empty model (1) as defined by a *p* < 0.05 in the analysis of deviance, the outcome analysis was continued. A stepwise variable reduction based on the Aikake information criterion was performed on the full model and the resulting list of covariates was compared between outcomes. A consolidated, minimal list of common covariates was created, and final models were calculated. Our multivariate logistic model consisted of the following covariates: AF on admission, known AF on admission, new-onset AF, age ≥ 79 years, BMI, sO_2_/FiO_2_, prevalence/incidence of COVID-19 during hospitalization, autoimmune disorder, immunosuppressive therapy, angiotensin-converting enzyme inhibitors (ACEI), sex, aspirin use and ageusia. A full list of the mentioned parameters with calculated odds ratios (OR) can be found in Figure 1, Figure 2, Figure 3 and Figure 4.

For survival analysis, a Cox proportional hazards model was performed with a further reduced set of covariates. The proportional hazards assumption was checked using the cox.zph function of the survival package. Calculations were performed using R version 4.1.3 with the survival and ROCit packages. A *p*-value for any used statistic test of <0.05 was considered significant.

## 3. Results

A total of 646 patients including 379 (59%) men were investigated in this study. The median age was 70 years (59–80). Amongst those 646 patients, 177 (27.4%) were transferred to the IMC/ICU and 76 (11.8%) were intubated during hospitalization. Ninety patients (13.9%) died. The remaining patients (*n* = 556; 86.1%) survived and were discharged from the hospital. Baseline characteristics can be obtained from Table 1. The median time of hospitalization was 9 (±9) days. The mean follow-up time was 278 (±90) days. The calculated age cut-off for the increased risk of in-hospital death was 79 years. A total of 116 patients (18%) had AF on admission of which 34 (29%) had newly diagnosed AF. Compared with patients with COVID-19 without AF, those with AF were significantly older (80 years, IQR 72–87 years vs. 67 years, IQR 57–78 years; *p* < 0.001). Median duration of hospitalization was 9 days (IQR 5–16 days) in the AF group and 6 days (IQR 4–10 days) in the no AF group. Notably, high sensitivity Troponin T (TnT-hs) levels and N-terminal pro-B-type natriuretic peptide (NT-proBNP) levels were significantly higher in COVID-19 patients with AF than in COVID-19 patients without AF (Table 1). Preexisting medical conditions such as CAD (22.4% vs. 10.0%; *p* < 0.001), cardiomyopathy (27.6% vs. 6.0%; *p* < 0.001) and kidney disease (25.9% vs. 12.7%; *p* < 0.001) were more often present in the cohort with AF than in the no AF cohort. Vital signs on admission did not differ between AF groups. Further information is given in Table 1. Compared with patients with COVID-19 without AF on admission, those with AF were transferred to the IMC/ICU significantly more frequently (35.3% vs. 25.7%; *p* = 0.034). In-hospital mortality was significantly higher among those patients with AF than among those without AF (28.4% vs. 10.8%; *p* < 0.001). However, after correction for confounders using our multivariate logistic model, this association was no longer detectable.

### 3.1. Outcomes

**Figure 5:**
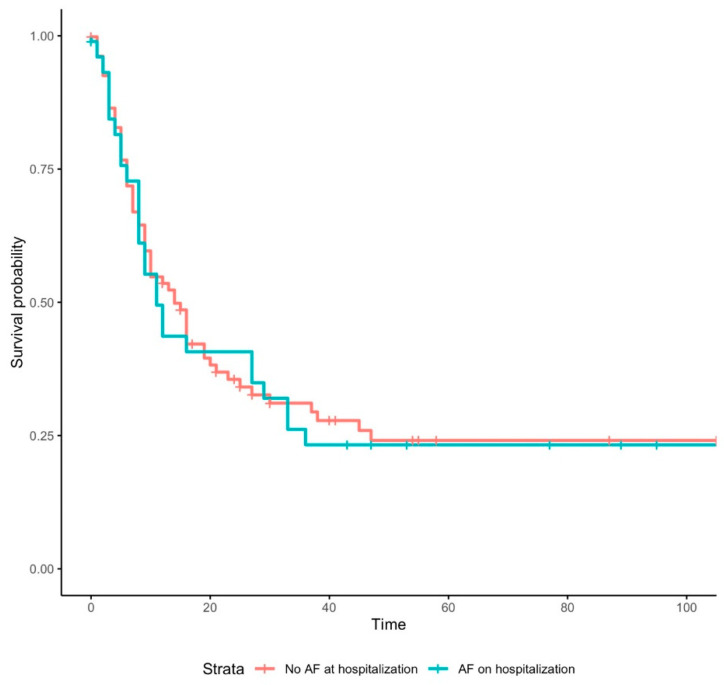
Kaplan–Meier survival curve for “In-Hospital Death” in patients with COVID-19 with and without atrial fibrillation. Abbreviations: AF = atrial fibrillation.

**Figure 6:**
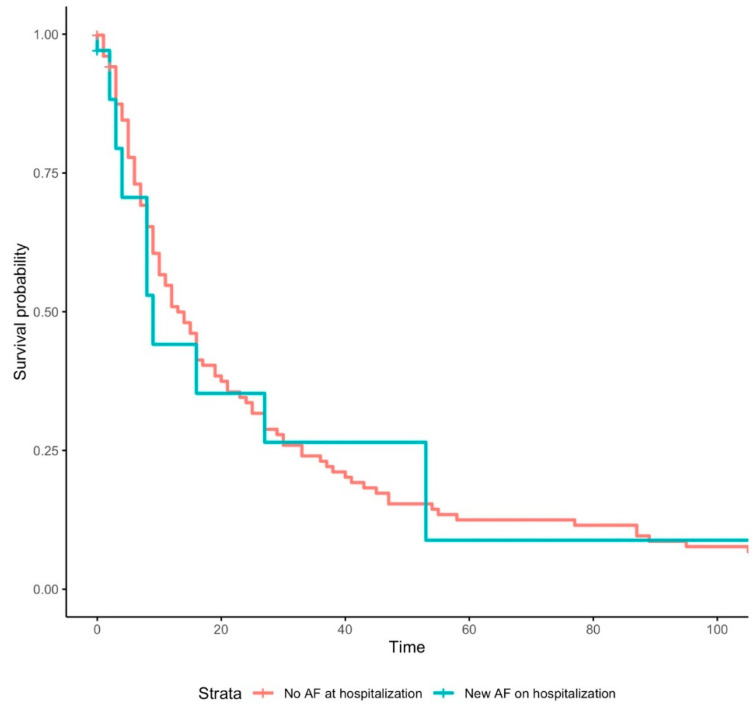
Kaplan–Meier survival curve for “Long-Term Mortality” in patients with COVID-19 with no atrial fibrillation and newly diagnosed atrial fibrillation. Abbreviations: AF = atrial fibrillation.

In our multivariate logistic model, after correction for potential confounders, AF on admission demonstrated no significant results with respect to any of the outcomes. Indeed, there was no association of new-onset AF with in-hospital mortality, number of rehospitalization or long-term mortality. New-onset AF, on the other hand, increased the risk of invasive ventilation significantly with an OR of 3.5 (*p* = 0.01). However, there was no association with in-hospital mortality (Figure 5), number of rehospitalizations, or long-term mortality (Figure 6). The odds for invasive ventilation were decreased when patients were on ACEI (OR: 0.04; *p* = 0.04), were older (≥79 years; OR: 0.31; *p* < 0.01) and were female (OR: 0.21; *p* < 0.01).

Predictive factors for in-hospital mortality were age ≥ 79 years (OR: 9.53; *p* < 0.01) and immunosuppressive therapy (OR: 4.3; *p* = 0.01). The risk for long-term mortality was highest in individuals aged ≥79 years (OR: 10.81; *p* < 0.01), which was followed by immunosuppressive therapy (OR: 4.14; *p* < 0.01).

### 3.2. Subgroup Analysis

The subgroup analysis comparing new-onset AF vs. patients with preexisting AF can be found in Table 2. Among patients with AF on admission, 34 (29.3%) had newly diagnosed AF. These tend to be younger and were less likely to have underlying cardiomyopathies or chronic kidney disease than patients with known AF (Table 2). Notably, acute intensive care was more frequently required in patients with COVID-19 and new-onset AF vs. those with known AF (58.8% vs. 25.6%; *p* = 0.001). In-hospital mortality did not differ between the two groups (26.5% vs. 29.3%; *p* = 0.76).

## 4. Discussion

Within the margins of our study, new-onset AF in patients presenting with COVID-19 increased the risk of transfer to the IMC/ICU and invasive ventilation. Nevertheless, AF was not associated with an increased in-hospital mortality after adjusting for potential confounders. These observations are in line with the results of a large US cohort [28]. In addition, we also did not observe an association between AF and long-term outcomes such as number of rehospitalizations or long-term mortality after hospital discharge.

These results demonstrate the significance of AF as a prognostic marker with regard to the acute clinical trajectory of COVID-19 after presentation to the emergency room. This is particularly important during periods when there is a high incidence of COVID-19, during which resources and time are limited and prognostic features such as AF might facilitate treatment decisions. Interestingly, also no effect on in-hospital mortality was demonstrated. While AF in the context of COVID-19 has been reported to be associated with a poor prognosis during hospitalization, data on in-hospital mortality are conflicting. While multiple studies suggested increased in-hospital mortality in patients with AF and COVID-19, the range of calculated risk varied. Moreover, in those studies, no statistical significance was identified after correction for confounders [29,30].

The focus of our study was to evaluate the clinical long-term outcome of patients with new-onset AF and COVID-19. No association between AF and long-term out-of-hospital mortality was identified, suggesting that AF is more likely to reflect severe disease and inflammation during acute illness, where it was associated with a higher risk for invasive ventilation. The fact that patients with COVID-19 and AF were older and had more comorbidities supports the hypothesis that age may contribute to higher levels of proinflammatory cytokines, which might trigger AF and therefore increase the risk for mortality in patients with COVID-19 [31]. This might also explain why older age seemed protective of invasive ventilation as invasive therapy might be used more restrictive in the elderly with comorbidities and poor prognosis.

ACEI intake was found to be a risk factor for poor clinical trajectory. ACEI do not influence ACE2 directly. However, the ACE2 gene and protein expression might be influenced by RAAS inhibitors. As a result, multiple hypotheses regarding this topic were discussed with controversial data [32]. In the end, an extensive meta-analysis by Baral et al. confirmed that the use of ACEI or ARBs does not increase the risk of mortality but may even have a protective effect [33]. While our data is contradicting this notion, it has to be mentioned that our study was not designed to evaluate these effects. Hence, these results have to be interpreted with caution. TnT-hs and NT-proBNP were also significantly higher in patients suffering from AF on admission. This is especially interesting as increased TnT-hs levels were described in patients suffering from COVID-19. It might be hypothesized that AF is responsible for a significant fraction of elevated TnT-hs in this population, which was already discussed in a study by Iorio et al. [34]. There was no difference in D-dimer levels between COVID-19 patients with and without AF. Interestingly, the rate of pulmonary embolism was significantly higher in the non-AF group compared to the AF group, which was most likely due to the already existing intake of oral anticoagulation medication in the AF group.

Newly diagnosed AF was associated with an increased risk of invasive ventilation, while preexisting AF was not. This is especially important as patients with new-onset AF tend to be younger and healthier in comparison with patients with preexisting AF, partially explaining that there was no association between AF-status and in-hospital mortality. Hence, the new development of AF by itself seemed to be a risk factor for poor prognosis and might reflect an even more severe inflammatory state. It is known that AF worsens the outcome in critically ill patients [35,36,37,38]. Accordingly, Musikantow et al. demonstrated similar rates of AF in COVID-19 and influenza and therefore concluded that AF is not specifically linked to COVID-19 but is a generalized response to systemic inflammation in viral diseases [39]. Similarly, a large Taiwanese study showed that an influenza infection is a risk factor for developing AF and that a vaccination can reduce this risk to a baseline level [40]. However, it is yet to be determined if the onset of AF in COVID-19 patients is a mere sign of morbidity or an independent factor for a poorer clinical trajectory. A causal relation between COVID-19 and the development of AF could not yet be determined, but several promoting metabolic pathways triggered by SARS-CoV-2 were described. Systemic inflammation with altered electrophysiology (calcium homeostasis, connexins) and ultimate atrial structural remodeling (cytokine mediated myocyte apoptosis, metalloproteinase and fibroblast activation) are hypothesized as potential causes [41]. As such, an additive effect of the virus is plausible. The incidence of new-onset AF was indeed comparable to the incidence of non-COVID-19 infections, supporting the hypothesis that AF might be triggered by any kind of inflammation and not specifically by COVID-19 [42].

### Limitations

Our study has several limitations. First, the single-center design limits our ability to draw a generalized conclusion about COVID-19 as the pandemic is extremely dynamic, and the clinical trajectory is also dependent on local factors such as healthcare infrastructure, hospitals, as well as health worker capacity, regional breakouts and treatment options. In addition, our population included data from the first wave of COVID-19 in 2020. Hence, results might not be transferable to other variants of SARS-CoV-2 and vaccinated individuals.

## 5. Conclusions

New-onset of AF is associated with a poor clinical trajectory in patients suffering from COVID-19. However, this does not translate to long-term outcomes as AF was not associated with increased mortality or rehospitalization after convalescence of COVID-19.

## Figures and Tables

**Table 1:** Characteristics of COVID-19 Patients with and without Atrial Fibrillation.

	Total Population (*n* = 646)	Atrial Fibrillation (*n* = 116)	w/o Atrial Fibrillation (*n* = 530)	*p*-Value
**Demographics**				
Female sex, no./total no. (%)	267/646 (41.3)	39/116 (33.6)	228/530 (43.0)	0.06
Age, y—median (IQR)	70 (59–80) *n* = 646	80.0 (72.0–87.0) *n* = 116	67.0 (57.0–78.0) *n* = 530	<0.001
BMI, kg/m^2^—median (IQR)	27 (24–31) *n* = 646	26.7 (23.7–29.6) *n* = 116	27.5 (24.3–31.2) *n* = 530	0.10
**Symptoms, no./total no. (%)**				
Fever	317/646 (49.1)	47/116 (40.5)	270/530 (50.9)	0.042
Cough	351/646 (54.3)	53/116 (45.7)	298/530 (56.2)	0.039
Diarrhea	163/646 (25.2)	27/116 (23.3)	136/530 (25.7)	0.59
Myalgia	101/646 (15.6)	11/116 (9.5)	90/530 (17.0)	0.044
Ageusia	27/646 (4.2)	1/116 (0.9)	26/530 (4.9)	0.049
**Vital signs, median (IQR)**				
Systolic blood pressure, mmHg	132.0 (119.0–146.0) *n* = 646	130.5 (113.5–146.0) *n* = 116	133.0 (120.0–145.3) *n* = 530	0.54
Diastolic blood pressure, mmHg	78.0 (67.5–86.0) *n* = 646	75.0 (60.0–87.0) *n* = 116	78.0 (68.0–85.3) *n* = 530	0.14
Heart rate, bpm	84.0 (74.0–95.0) *n* = 646	84.0 (72.3–93.0) *n* = 116	84.0 (74.0–96.0) *n* = 530	0.74
Temperature, °C	37.0 (36.3–37.9) *n* = 646	36.9 (36.2–37.9) *n* = 116	37 (36.3–37.9) *n* = 530	0.27
Blood oxygen saturation, %	93.7 (91.5–95.8) *n* = 451	93.8 (90.8–96.0) *n* = 85	93.7 (91.7–95.8) *n* = 366	0.85
pO_2_, mmHg	67.3 (60.5–76.4) *n* = 451	68.5 (60.5–81.7) *n* = 85	67.0 (60.5–75.8) *n* = 366	0.41
pCO_2_, mmHg	31.9 (28.5–34.9) *n* = 452	30.9 (27.5–35.6) *n* = 85	32.1 (28.8–34.8) *n* = 367	0.20
P/F ratio	383.3 (321.9–400.0) *n* = 508	375.0 (296.9–400.0) *n* = 89	383.3 (339.3–400.0) *n* = 419	0.12
**Laboratory findings, median (IQR)**				
Troponin T hs, ng/L	13.8 (7.9–35.5) *n* = 147	35.7 (16.4–60.4) *n* = 35	10.6 (6.9–25.2) *n* = 112	<0.001
Creatine kinase, U/L	98.5 (59.3–185.5) *n* = 180	113.0 (72.0–208.0) *n* = 43	85.0 (55.0–176.0) *n* = 137	0.027
NT-proBNP, ng/L	772.5 (207.3–3623) *n* = 94	3402.0 (2000.0–6908.5) *n* = 26	342.0 (154.8–1495.5) *n* = 68	<0.001
CRP, mg/L	58.1 (24.0–111.4) *n* = 623	60.9 (23.2–113.9) *n* = 112	57.3 (24.0–110.6) *n* = 511	0.49
PCT, ng/mL	0.12 (0.07–0.25) *n* = 526	0.17 (0.10–0.49) *n* = 96	0.11 (0.07–0.21) *n* = 430	<0.001
Creatinine, µmol/L	84.0 (67.0–111.0) *n* = 621	102.5 (82.3–139.5) *n* = 112	82.0 (64.0–105.0) *n* = 509	<0.001
D-Dimer, µg FEU/L	493.5 (297.5–1063.8) *n* = 448	460.0 (211.8–1377.3) *n* = 76	499.5 (300.8–1027.8) *n* = 372	0.43
**Cardiovascular risk factors, no./total no. (%)**				
Hypertension	302/644 (46.9)	71/116 (61.2)	231/528 (43.8)	<0.001
Diabetes mellitus	159/644 (24.6)	35/116 (30.2)	124/528 (23.5)	0.13
Dyslipidemia	91/644 (14.1)	26/116 (22.4)	65/528 (12.3)	0.005
Cardiovascular family history	9/644 (1.4)	3/116 (2.6)	6/528 (1.1)	0.23
**Coexisting medical condition, no./total no. (%)**				
Coronary artery disease	79/646 (12.2)	26/116 (22.4)	53/530 (10.0)	<0.001
Hematologic disorders	60/645 (9.2)	16/116 (13.8)	44/529 (8.3)	0.07
Cancer	70/646 (10.8)	15/116 (12.9)	55/530 (10.4)	0.42
Cardiomyopathy	64/646 (9.9)	32/116 (27.6)	32/530 (6.0)	<0.001
Chronic kidney disease	97/645 (15.0)	30/116 (25.9)	67/529 (12.7)	<0.001
Chronic infections	7/646 (1.1)	2/116 (1.7)	5/530 (0.9)	0.46
Autoimmune disorders	24/646 (3.7)	3/116 (2.6)	21/530 (4.0)	0.48
**Medication on admission, no./total no. (%)**				
ACE inhibitor	245/646 (37.9)	49/116 (42.2)	196/530 (37.0)	0.29
Beta-blocker	158/646 (24.5)	57/116 (49.1)	101/530 (19.1)	<0.001
Calcium-channel antagonist	121/646 (18.7)	31/117 (26.7)	90/530 (17.0)	0.015
Statin	187/646 (28.9)	51/116 (44.0)	136/530 (25.7)	<0.001
Aspirin	116/646 (18.0)	16/117 (13.8)	100/530 (18.9)	0.20
Anticoagulation	141/646 (21.8)	76/116 (65.5)	65/530 (12.3)	<0.001
Immunosuppressive therapy	32/646 (5.0)	2/116 (1.7)	30/530 (5.7)	0.08
**Complications/management, no./total no. (%)**				
Intensive care (IMC/ICU)	177/646 (27.4)	41/116 (35.3)	136/530 (25.7)	0.034
Catecholamine use	59/646 (9.1)	12/116 (10.3)	47/530 (8.9)	0.62
Invasive ventilation	76/645 (11.8)	16/116 (13.8)	60/529 (11.3)	0.46
Noninvasive ventilation	75/645 (11.6)	13/116 (11.2)	62/529 (11.7)	0.88
In-hospital mortality	90/646 (13.9)	33/116 (28.4)	57/530 (10.8)	<0.001
Poor clinical trajectory	224/646 (34.7)	58/116 (50.0)	166/530 (31.3)	<0.001
DVT	6/646 (0.9)	3/116 (2.6)	3/530 (0.6)	0.040
Pulmonary embolism	31/644 (4.8)	1/115 (0.9)	30/529 (5.7)	0.029
ARDS	37/636 (5.8)	10/114 (8.8)	27/522 (5.2)	0.14

Values are results of Chi^2^ test in nominal scaled variables or Mann–Whitney U-test in nonparametric continuous variables. Poor clinical trajectory is a composite of IMC/ICU transfer or in-hospital mortality. Abbreviations: ACE = angiotensin-converting enzyme; ARDS = acute respiratory distress syndrome; BMI = Body mass index; NT-proBNP = N-terminal pro-B-type natriuretic peptide; CRP = C-reactive protein; DVT = deep vein thrombosis; FEU = fibrinogen-equivalent units; ICU = intensive care unit; IMC, intermediate care unit; IQR = interquartile range; pCO_2_ = partial blood carbon dioxide pressure; PCT = procalcitonin; P/F = arterial pressure of oxygen and fraction of inspired oxygen ratio; pO_2_ = partial blood oxygen pressure.

**Table 2:** Characteristics of COVID-19 Patients with New-onset and Preexisting AF.

	New-Onset AF (*n* = 34)	Preexisting AF (*n* = 82)	*p*-Value
**Demographics**			
Female sex, no./total no. (%)	11/34 (32.4)	28/82 (34.1)	0.85
Age, y—median (IQR)	75.5 (66.0–87.3) *n =* 34	81.0 (73.8–87.0) *n =* 82	0.059
BMI, kg/m^2^—median (IQR)	26.6 (22.8–30.3) *n =* 34	27 (23.8–29.7) *n =* 82	0.74
**Symptoms, no./total no. (%)**			
Fever	16/34 (47.1)	31/82 (37.8)	0.36
Cough	17/34 (50.0)	36/82 (43.9)	0.55
Diarrhea	9/34 (26.5)	18/82 (22.0)	0.60
Myalgia	2/34 (5.9)	9/82 (11.0)	0.39
Ageusia	1/34 (2.9)	0/82 (0.0)	0.12
**Vital signs, median (IQR)**			
Systolic blood pressure, mmHg	132.5 (120.0–154.3) *n =* 34	129.0 (112.0–146.0) *n =* 82	0.35
Diastolic blood pressure, mmHg	80.0 (59.8–89.3) *n =* 34	73.5 (59.8–86.3) *n =* 82	0.53
Heart rate, bpm	84.5 (75.3–98.5) *n =* 34	83.5 (71.8–92.3) *n =* 82	0.66
Temperature, °C	37.0 (36.0–38.0) *n =* 34	36.9 (36.4–37.9) *n =* 82	0.87
Blood oxygen saturation, %	93.8 (90.7–95.9) *n =* 27	94.1 (90.7–96.4) *n =* 58	0.96
pO_2_, mmHg	68.3 (60.9–79.4) *n =* 27	68.6 (60.4–82.7) *n =* 58	0.78
pCO_2_, mmHg	29.8 (26.7–35.3) *n =* 27	31.5 (27.7–35.8) *n =* 58	0.54
P/F ratio	333.3 (284.4–383.3) *n =* 27	387.5 (301.6–401.0) *n =* 62	0.14
**Laboratory findings, median (IQR)**			
Troponin T hs, ng/L	25.4 (13.2–60.9) *n =* 13	37.9 (17.5–58.1) *n =* 22	0.91
Creatine kinase, U/L	121.0 (67.0–504.8) *n =* 18	108.0 (77.0–192.0) *n =* 25	0.56
NT-proBNP, ng/L	2970.0 (1170.5–10,061.5) *n =* 9	3653.0 (2250.5–6523.0) *n =* 17	0.87
CRP, mg/L	70.6 (31.7–170.0) *n =* 33	59.3 (20.2–103.8) *n =* 79	0.19
PCT, ng/mL	0.22 (0.12–0.59) *n =* 31	0.14 (0.09–0.49) *n =* 65	0.26
Creatinine, µmol/L	92.0 (71.5–117.0) *n =* 33	114.0 (89.0–144.0) *n =* 79	0.009
D-dimer, µg FEU/L	575.5 (227.8–3783.8) *n =* 28	395.5 (211.0–779.0) *n =* 48	0.12
**Cardiovascular risk factors, no./total no. (%)**			
Hypertension	19/34 (55.9)	52/82 (63.4)	0.45
Diabetes mellitus	14/34 (41.2)	21/82 (25.6)	0.10
Dyslipidemia	8/34 (23.5)	18/82 (22.0)	0.85
Adipositas	6/34 (17.6)	16/82 (19.5)	0.82
Cardiovascular family history	1/34 (2.9)	2/82 (2.4)	0.88
**Coexisting medical condition, no./total no. (%)**			
Coronary artery disease	7/34 (20.6)	19/82 (23.2)	0.76
Hematologic disorders	4/34 (11.8)	12/82 (14.6)	0.68
Cancer	2/34 (5.9)	13/82 (15.9)	0.15
Cardiomyopathy	5/34 (14.7)	27/82 (32.9)	0.046
Chronic kidney disease	2/34 (5.9)	28/82 (34.1)	0.002
Chronic infections	1/34 (2.9)	1/82 (1.2)	0.52
Autoimmune disorders	1/34 (2.9)	2/82 (2.4)	0.88
**Medication on admission, no./total no. (%)**			
ACE inhibitor	14/34 (41.2)	35/82 (42.7)	0.88
Beta blocker	11/34 (32.4)	46/82 (56.1)	0.020
Calcium-channel antagonist	9/34 (26.5)	22/82 (26.8)	0.97
Statin	13/34 (38.2)	38/82 (46.3)	0.42
Aspirin	10/34 (29.4)	6/82 (7.3)	0.002
Anticoagulation	8/34 (23.5)	68/82 (82.9)	<0.001
Immunosuppressive therapy	0/34 (0.0)	2/82 (2.4)	0.36
**Complications/management, no./total no. (%)**			
Intensive care (IMC/ICU)	20/34 (58.8)	21/82 (25.6)	0.001
Catecholamine use	3/34 (8.8)	9/82 (11.0)	0.73
Invasive ventilation	9/34 (26.5)	7/82 (8.5)	0.011
Noninvasive ventilation	5/34 (14.7)	8/82 (9.8)	0.44
In-hospital mortality	9/34 (26.5)	24/82 (29.3)	0.76
Poor clinical trajectory	25/34 (73.5)	33/82 (40.2)	0.001
DVT	3/34 (8.8)	0/82 (0.0)	0.006
Pulmonary embolism	1/34 (2.9)	0/82 (0.0)	0.12
ARDS	8/34 (23.5)	2/82 (2.5)	<0.001

Values are results of Chi^2^ test in nominal scaled variables, or Mann–Whitney U-test in nonparametric continuous variables. Poor clinical trajectory is a composite of IMC/ICU transfer or in-hospital mortality. Abbreviations: ACE = angiotensin-converting enzyme; ARDS = acute respiratory distress syndrome; BMI = Body mass index; NT-proBNP = N-terminal pro-B-type natriuretic peptide; CRP = C-reactive protein; DVT = deep vein thrombosis; FEU = fibrinogen-equivalent units; ICU = intensive care unit; IMC, intermediate care unit; IQR = interquartile range; pCO_2_ = partial blood carbon dioxide pressure; PCT = procalcitonin; P/F = arterial pressure of oxygen and fraction of inspired oxygen ratio; pO_2_ = partial blood oxygen pressure.

## Data Availability

The data presented in this study is available on request from the corresponding author. The data is not publicly available due to patient’s confidentiality.

## Abbreviations

ACE	Angiotensin-converting enzyme
ACE2	Angiotensin-converting enzyme 2
ACEI	Angiotensin-converting enzyme inhibitor
AF	Atrial fibrillation
ARB	Angiotensin-receptor blocker
ARDS	Acute respiratory distress syndrome
BMI	Body mass index
CAD	Coronary artery disease
CMP	Cardiomyopathy
COVID-19	Coronavirus disease 2019
CRP	C-reactive protein
CV	Cardiovascular
DVT	Deep vein thrombosis
ECG	Electrocardiogram
ER	Emergency room
ICU	Intensive care unit
IMC	Intermediate care unit
IQR	Interquartile range
MD	Medical doctor
NT-proBNP	N-terminal pro-B-type natriuretic peptide
OR	Odds ratio
PCR	Polymerase chain reaction
PCT	Procalcitonin
ROC	Receiver operating characteristic
SARS-CoV-2	Severe acute respiratory syndrome coronavirus type 2
sO_2_	Arterial oxygen saturation
TnT-hs,	Troponin T high sensitivity
US	United States
